# Early Detection and Surveillance of the SARS-CoV-2 Variant BA.3.2 — Worldwide, November 2024–February 2026

**DOI:** 10.15585/mmwr.mm7510a1

**Published:** 2026-03-19

**Authors:** Mila Shakya, Kevin C. Ma, Laura J. Hughes, Casey Smith, Lydia J. Atherton, Alexandria B. Boehm, Peter W. Cook, Daniel M. Cornforth, Meredith Gardner, Iryna V. Goraichuk, Jennifer L. Harcourt, Heather Hicks, Marc Johnson, Anna Kelleher, Heather E. Reese, Susanna J. Sabin, Teresa C. Smith, Azaibi Tamin, Marlene K. Wolfe, Benjamin J. Silk, Clinton R. Paden, Natalie Thornburg, Adam MacNeil

**Affiliations:** ^1^Coronavirus and Other Respiratory Viruses Division, National Center for Immunization and Respiratory Diseases, CDC; ^2^Epidemic Intelligence Service, CDC; ^3^Department of Civil and Environmental Engineering, Stanford University, Stanford, California; ^4^Division of Infectious Disease Readiness and Innovation, National Center for Emerging and Zoonotic Infectious Diseases, CDC; ^5^ASRT, Inc., Smyrna, Georgia; ^6^School of Medicine, University of Missouri, Columbia, Missouri; ^7^Division of Global Migration Health, National Center for Emerging and Zoonotic Infectious Diseases, CDC; ^8^Rollins School of Public Health, Emory University, Atlanta, Georgia.

**Summary**

**What is already known about this topic?**

CDC tracks SARS-CoV-2 variants internationally using digital public health surveillance and in the United States using genomic surveillance, including wastewater and traveler-based surveillance. The highly divergent SARS-CoV-2 variant BA.3.2 was first detected in a respiratory sample collected on November 22, 2024, in South Africa.

**What is added by this report?**

As of February 11, 2026, BA.3.2 had been reported in 23 countries. Detections began increasing in September 2025. In the United States, BA.3.2 was detected in nasal swabs from four travelers, three airplane wastewater samples, clinical samples from five patients, and 132 wastewater samples from 25 U.S. states.

**What are the implications for public health practice?**

Monitoring the spread of BA.3.2 provides valuable information about the potential for this new SARS-CoV-2 lineage to evade immunity from a previous infection or vaccination.

## Abstract

The SARS-CoV-2 variant BA.3.2 was first identified in South Africa on November 22, 2024. BA.3.2 has approximately 70–75 substitutions and deletions in the gene sequence of the spike protein relative to JN.1 and its descendant, LP.8.1, the antigens used in the 2025–26 COVID-19 vaccines. CDC is using a multimodal SARS-CoV-2 genomic surveillance approach to monitor the emergence and spread of BA.3.2 and other SARS-CoV-2 variants internationally and within the United States. The first U.S. BA.3.2 detection occurred on June 27, 2025, through CDC’s Traveler-Based Genomic Surveillance program in a participant traveling to the United States from the Netherlands. The first U.S. detection of BA.3.2 in a clinical specimen collected from a patient was reported on January 5, 2026. As of February 11, 2026, BA.3.2 had been detected in voluntarily self-collected nasal swabs from four U.S. travelers, clinical samples from five patients, three airplane wastewater samples, and 132 wastewater surveillance samples from 25 states. BA.3.2 has been reported by at least 23 countries. SARS-CoV-2 continues to cause substantial morbidity and mortality worldwide. BA.3.2 mutations in the spike protein have the potential to reduce protection from a previous infection or vaccination. Continued genomic surveillance is needed to track SARS-CoV-2 evolution and determine its potential effect on public health.

## Introduction

Since the COVID-19 pandemic began in late 2019, new SARS-CoV-2 variants with mutations in the spike protein have continued to emerge, generating antigenic diversity and immune escape characteristics that necessitate periodic reevaluation and reformulation of the COVID-19 vaccine composition. The spike protein is the primary target of neutralizing antibodies generated from a previous infection or COVID-19 vaccination, and mutations in this protein can affect transmissibility and immune evasion. During December 2021, the B.1.1.529 (Omicron) variant, with approximately 32 spike mutations, began replacing pre-Omicron strains, evading neutralizing antibodies induced by previous infection or vaccination and resulting in a surge in COVID-19 cases and hospitalizations (*1*). Subsequently, the XBB (2022) and BA.2.86 (2023) variants emerged, prompting updates to COVID-19 vaccines to include these new lineages (*2*–*4*). The BA.3.2 lineage descends from BA.3, which emerged and briefly cocirculated with BA.1 and BA.2 during late 2021 to 2022. BA.3.2 is characterized by enhanced in vitro immune escape, with reduced neutralization from human serum antibodies induced by current COVID-19 vaccines (*5*).

CDC monitors SARS-CoV-2 evolution through a multimodal genomic surveillance approach to track the emergence and spread of variants with substantial genetic changes across the United States and internationally (*3*,*4*). This report summarizes the identification and circulation of the BA.3.2 variant and associated sublineages in the United States and worldwide during November 2024–February 2026.

## Methods

### Data Sources

CDC tracks SARS-CoV-2 variants internationally using digital public health surveillance (*4*), which includes monitoring sequences that are submitted to open-access sequence repositories such as the Global Initiative on Sharing All Influenza Data and the National Center for Biotechnology Information (NCBI) GenBank* (*6*), as well as surveying media, GitHub, preprint databases, and social media platforms for variant reports. In the United States, CDC integrates sequence data from the national SARS-CoV-2 genomic surveillance program (Supplementary Figure), the Traveler-Based Genomic Surveillance (TGS) program,^†^ and the National Wastewater Surveillance System (NWSS). This multimodal genomic surveillance approach has been described previously (*4*). The national genomic surveillance program combines sequencing data from the National SARS-CoV-2 Strain Surveillance program and sequence repositories to estimate variant proportions over time. The TGS program tests airplane wastewater samples and self-collected nasal swabs from volunteer international travelers to detect pathogens, including emerging SARS-CoV-2 variants; subsequent sequences are uploaded to the TGS NCBI BioProject. Wastewater-based surveillance was conducted through NWSS, which monitors approximately 1,300 U.S. wastewater sites, and WastewaterSCAN^§^ (*7*), which monitors 150 sites; both conduct pathogen detection and SARS-CoV-2 variant characterization. The resulting sequences were uploaded to BioProject PRJNA747181 and PRJNA957477, respectively.

### Genetic Analyses

CDC analyzed BA.3.2 genome sequences reported through global open-access sequence repositories and those from isolates collected and sequenced in the United States; complete sequences were selected for representation in phylogenetic analysis. BA.3.2 lineage assignments were confirmed using Nextclade (version 3.18.1; Nextstrain). BA.3.2 in wastewater samples was identified using Freyja (version 1.5.2; GitHub) and CoOccurence adJusted Analysis and Calling (version 0.9.3; GitHub).^¶^ A subset of BA.3.2 sequences from the United States (nine) and representative international sequences (seven) with good quality (as defined in Nextclade) were selected for spike protein mutation and phylogenetic analyses. Spike protein genome sequence substitutions, deletions, and insertions in BA.3.2 sequences were calculated with reference comparison to LP.8.1, one of the antigens used in 2025–2026 mRNA COVID-19 vaccines. Spike gene regions from BA.3.2, LP.8.1, and other representative SARS-CoV-2 strains, were aligned with the multiple alignment using fast Fourier transform (MAFFT) program (version 7.490; Kazutaka Katoh), an alignment program for multiple amino acid or nucleotide sequences. An unrooted maximum likelihood tree was inferred using PhyML (version 3.3.20180621; GitHub), a software package that analyzes alignments of nucleotide or amino acid sequences in a phylogenetic framework, and was visualized in Interactive Tree of Life (version 7.4.1; biobyte solutions), an online tool for the display and management of phylogenetic trees.

### Epidemiologic Analyses

All confirmed BA.3.2 detections were aggregated by country and epidemiologic week. International and U.S. BA.3.2 detections were mapped by the earliest sample collection date. The interval between dates of SARS-CoV-2 specimen collection and reporting was calculated. The prevalence of BA.3.2 detections was estimated with 95% CIs calculated using an exact binomial test. Analyses were conducted using R (version 4.5.0; R Foundation). Data were current as of February 11, 2026. This activity was reviewed by CDC, deemed not research, and conducted consistent with applicable federal law and CDC policy.** Isolation of SARS-CoV-2 from anonymous volunteers was deemed research not involving human subjects by CDC.

## Results

### Characteristics of the BA.3.2 Lineage

**FIGURE 1:**
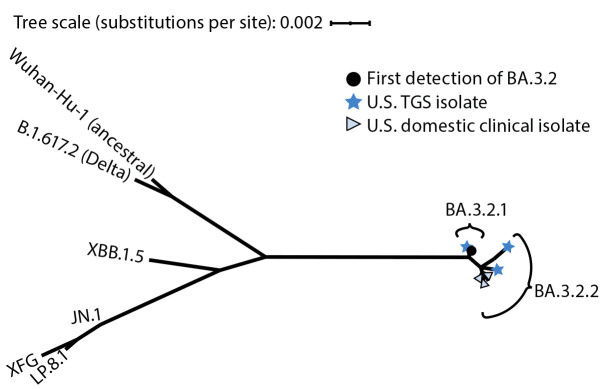
Phylogenetic tree* of the SARS-CoV-2 spike glycoprotein gene from selected U.S.^†^ and global^§^ BA.3.2 isolates^¶^ and reference strains — 2020–2026 **Abbreviation: **MAFFT = multiple alignment using fast Fourier transform. * Spike gene regions from 16 BA.3.2 isolates and representative historical SARS-CoV-2 lineages were aligned using MAFFT, and an unrooted maximum likelihood tree was inferred using PhyML (version 3.3.20180621; GitHub). Branch lengths represent genetic distance between sequences as measured by estimated numbers of nucleotide substitutions per site. ^†^ U.S. BA.3.2 isolates comprise the first three detections in nasal swabs from international travelers (from Japan, Kenya, and the Netherlands) participating in the TGS program and the first three U.S. clinical detections. ^§^ The global BA.3.2 isolates include the first BA.3.2 detection, which was isolated from a nasopharyngeal swab from a boy aged 5 years by investigators in South Africa. Evolution and Viral Properties of the SARS-CoV-2 BA.3.2 Subvariant | medRxiv ^¶^ Includes BA.3.2.1 (and descendants RD.1, RD.1.1 [contains a V227L mutation in the spike protein] and RD.1.2), and BA.3.2.2 (and descendants RE.1 and RE.2).

BA.3.2 represents a new lineage of SARS-CoV-2, genetically distinct from the JN.1 lineages (including LP.8.1 and XFG) that have circulated in the United States since January 2024 (Figure 1). Relative to LP.8.1, BA.3.2 has 20 receptor-binding domain differences and 35 N-terminal domain differences, with deletions spanning sites 136–147 and 243–244 and an insertion of four amino acids after site 214 (Supplementary Table 1). Phylogenetically, the magnitude of spike gene divergence compared with LP.8.1 is larger than the corresponding difference between JN.1 and XBB.1.5, the target antigen of 2023–2024 COVID-19 vaccines (Figure 1). BA.3.2 comprises two major branches, BA.3.2.1 and BA.3.2.2. Relative to BA.3.2.1, BA.3.2.2 has the following substitutions: K356T, A575S, R681H, and R1162P (Supplementary Table 1). BA.3.2 spike sequences from U.S. isolates are distributed throughout the spike gene phylogeny, consistent with multiple independent domestic introductions (Figure 1).

### Global BA.3.2 Detections

**FIGURE 2:**
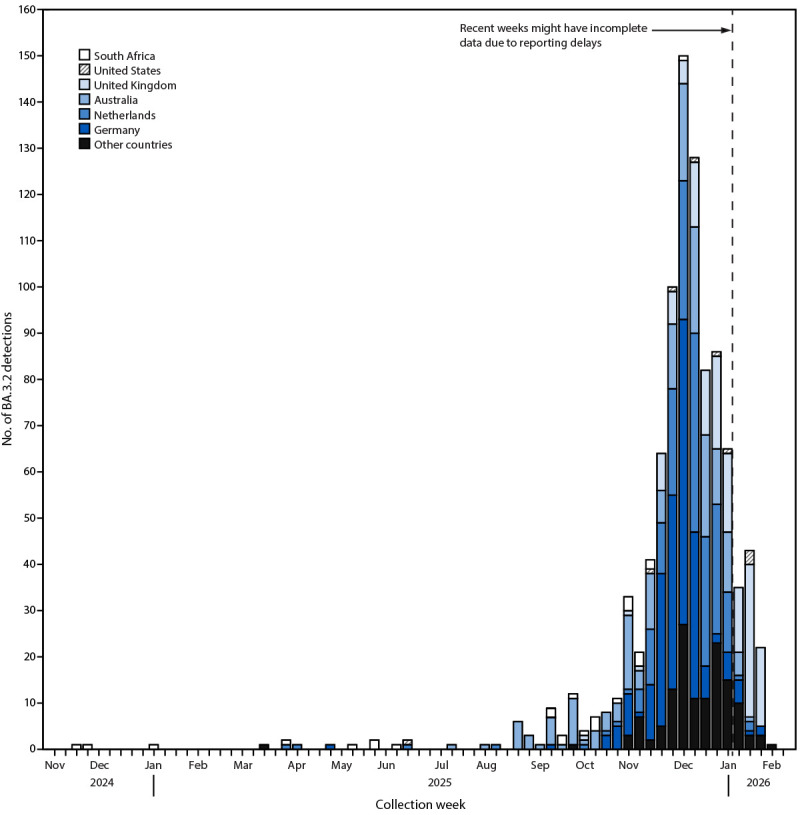
SARS-CoV-2 variant BA.3.2 detections,* by week^†^ and country of origin^§^ — worldwide, November 2024–February 2026^¶ ^ **Abbreviation: **TGS = Traveler-Based Genomic Surveillance. * BA.3.2 detections include national genomic surveillance detections reported to the Global Initiative on Sharing All Influenza Data by country of origin. Isolates from CDC’s TGS program are included under the total count for United States and not the countries of origin. U.S. TGS detected BA.3.2 in nasal swabs from participants traveling to the United States from Japan, Kenya, the Netherlands, and the United Kingdom. ^†^ Declines in the recent weeks should be interpreted with caution; case counts for the most recent weeks are incomplete because of delays between specimen collection and reporting. ^§^ Country of first detection (South Africa), the United States, and the four countries with the most detections are included separately. All other jurisdictions with BA.3.2 detections (Belgium, Canada, Czechia, China [specifically, Hong Kong], France, Italy, Luxembourg, Mozambique, New Zealand, Norway, Ireland, Singapore, South Korea, Slovenia, Spain, and Sweden) are combined and listed as other countries. ^¶^ Data current as of February 11, 2026.

**FIGURE 3:**
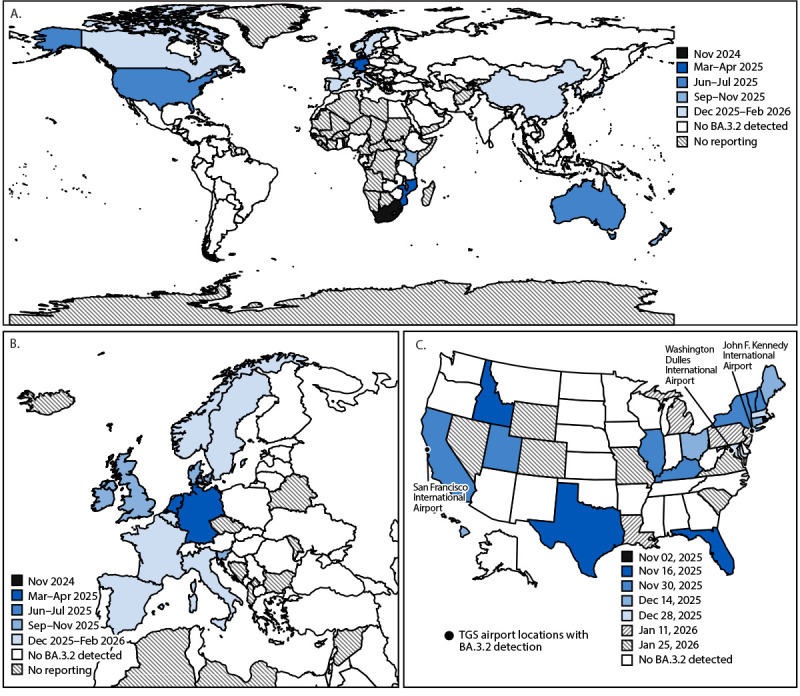
SARS-CoV-2 BA.3.2 detections,* by initial sample collection date^†^ — worldwide (A),^§^ Europe (B), and the United States (C),^¶^ November 2024–February 2026** **Abbreviations:** NWSS = National Wastewater Surveillance System; TGS = Traveler-Based Genomic Surveillance. * BA.3.2 detections include respiratory sample and wastewater detections. ^†^ Panels A and B were aggregated by month of sample collection; panel C was aggregated by start date of epidemiologic week. ^§^ All worldwide first detections were in respiratory surveillance samples, except for the United States, where the first detection was in a TGS program sample from a traveler from the Netherlands, and New Zealand, where the first detection was in a wastewater sample. Japan and Kenya represent the country of origin of TGS program samples collected in the United States; no BA.3.2 detections have been reported yet in either country. The following countries and U.S. freely associated states reported fewer than 50 sequences during 2024–25: Armenia, Bhutan, Bolivia, Burundi, Cuba, the Democratic Republic of the Congo, Ethiopia, the Federated States of Micronesia, Honduras, Iraq, Jordan, Kuwait, Latvia, Lebanon, Montenegro, Mozambique, Nepal, Nicaragua, North Macedonia, Palau, Saudi Arabia, Senegal, Serbia, Sri Lanka, Suriname, Uganda, Uruguay, Uzbekistan, and Zimbabwe. ^¶^ BA.3.2 was first detected in U.S. states through NWSS (California, Connecticut, Florida, Hawaii, Idaho, Maine, Maryland, Massachusetts, Missouri, Nevada, New Hampshire, New Jersey, New York, Pennsylvania, Rhode Island, South Carolina, Texas, Utah, Vermont, Virginia, and Wyoming), WastewaterSCAN (California, Louisiana, Maine, Massachusetts, Michigan, and Ohio), and genomic surveillance in four states. BA.3.2 was also detected in TGS program respiratory samples (San Francisco International Airport in California and John F. Kennedy International Airport in New York) and TGS program triturator and aircraft wastewater specimens (San Francisco International Airport and Washington Dulles International Airport in Virginia). ** Data current as of February 11, 2026.

The first BA.3.2 lineage sequence was detected in a respiratory sample collected on November 22, 2024, in South Africa (Figure 2) (Figure 3) (Supplementary Table 2). Investigators in South Africa isolated SARS-CoV-2 virus from a nasopharyngeal swab from a boy aged 5 years and designated the sequence as BA.3.2.1 under Pango nomenclature. On March 17, 2025, BA.3.2 was detected in Mozambique, followed by detections in the Netherlands (April 12) and Germany (April 29). After these initial detections, BA.3.2 detections were infrequent but began to increase in September 2025, with the highest number of detections reported during the week beginning December 7, 2025 (Figure 2). As of February 11, 2026, BA.3.2 had been detected in at least 23 countries, including four TGS program detections in U.S. travelers returning from Japan, Kenya, the Netherlands, and the United Kingdom (Supplementary Table 2) (Figure 3). During November 2025–January 2026, weekly BA.3.2 detections increased and reached approximately 30% of sequences reported in three European countries (Denmark, Germany, and the Netherlands), although the overall COVID-19 incidence was not substantially higher than the incidence during previous years. Among the initial BA.3.2 detections in each country, the median interval from specimen collection to sequence reporting for respiratory detections was 18 days (range = 3–123 days) (Supplementary Table 2).

### U.S. BA.3.2 Detections

On June 27, 2025, BA.3.2 was detected for the first time in the United States at the San Francisco International Airport in California in a respiratory sample collected from a TGS program participant who had traveled from the Netherlands (Figure 3) (Supplementary Table 2). CDC laboratories cultured and characterized the specimen, sharing the results with collaborators and depositing the data at Biodefense and Emerging Infection Research Resources.^††^ On November 11, 2025, the NWSS program detected BA.3.2 in a U.S. wastewater surveillance sample from Rhode Island.

The first three U.S. detections of BA.3.2 among patients were reported from samples collected during December 4, 2025–January 4, 2026, in three different U.S. states. BA.3.2 was detected in samples from two hospitalized older adult patients with comorbidities, including one patient admitted 4 days earlier for cardiac care, and a young child who received outpatient care. All patients survived.

As of February 11, 2026, BA.3.2 had been detected in five respiratory samples collected in four states. The prevalence of BA.3.2 detections among 2,579 total sequences in national surveillance collected during December 1, 2025–February 11, 2026, was 0.19% (95% CI = 0.06%–0.45%). BA.3.2 was also detected in four TGS program respiratory samples collected from international travelers, three airplane wastewater samples,^§§^ and 132 wastewater samples from 25 states through NWSS^¶¶^ and WastewaterSCAN.***

## Discussion

Since November 2024, the SARS-CoV-2 BA.3.2 lineage has been detected in Africa, Asia, Europe, North America, and Oceania. Numbers of reported detections have increased since September 2025; however, because many countries have limited genomic detection and surveillance capacities, these detections likely underrepresent the actual geographic extent of spread. Phylogenetic analyses have identified the emergence of two BA.3.2 sublineages (BA.3.2.1 and BA.3.2.2), indicating ongoing viral evolution (*8*). This analysis was facilitated through multimodal surveillance approaches, including global digital SARS-CoV-2 surveillance and enhanced U.S. surveillance among inbound international travelers, wastewater monitoring, and collaboration with public health laboratories, with data reported through February 11. As of March 12, BA.3.2 has been detected in nasal swabs from six U.S. travelers, three airplane wastewater samples, 29 patients, and 260 wastewater samples in 29 states and Puerto Rico. The prevalence of BA.3.2 detections among 5,238 sequences collected during December 1, 2025–March 12, 2026, is 0.55% (95% CI = 0.37%–0.79%).

The Omicron BA.2.86 lineage variant, first detected in 2023, had approximately 30 mutations in the spike protein relative to its predecessor, BA.2 (*3*). Despite low-level circulation, BA.2.86 had the potential to accumulate immune evasion mutations; subsequent acquisition of the L455S substitution in the BA.2.86 lineage likely conferred a selective advantage, leading to the emergence of JN.1, which then replaced the previously predominant XBB lineage viruses (*3*). This strain replacement event was the third observed in the United States; the first occurred during Omicron’s emergence during late 2021 and early 2022 (*1*), and the second corresponds to the replacement of BA.4/5 by XBB lineages during late 2022 and early 2023 (*3*). A strain replacement event has not been observed during 2024–25 or 2025–26.

The 2025–2026 LP.8.1-adapted mRNA COVID-19 vaccine demonstrates protection against currently predominant JN.1 strains but had the lowest antibody neutralization against BA.3.2 in a laboratory study of seven variants, potentially affecting vaccine-conferred protection, although observational data are also needed (*5*). Therefore, the observed increases in BA.3.2 detections, particularly in certain northern European countries, might be driven by substantial antibody evasion enabling infection of previously immune persons among populations in which previous variants circulated. However, in contrast to previous strain replacement events, BA.3.2 has not rapidly overtaken other variants; rather, in several European countries, BA.3.2 has cocirculated with various JN.1 descendant lineages with prevalences of approximately 10%–40%. Two laboratory studies found that BA.3.2.1 and BA.3.2.2 had substantially reduced angiotensin-converting enzyme 2 (ACE2) binding and lung cell entry compared with XFG and NB.1.8.1, potentially constraining its ability to rapidly become a dominant variant (*9*,*10*). Seasonal increases in COVID-19 transmission or further evolution of BA.3.2, potentially via compensatory mutations restoring ACE2 binding or infectivity, could enable broader circulation, although this has not yet been observed.

CDC’s complementary surveillance systems are widely used for tracking the global spread, domestic introduction, and genetic evolution of BA.3.2 and its sublineages. The TGS program detected BA.3.2 in a respiratory specimen from a traveler to the United States during late June 2025, marking the first documented U.S. entry of BA.3.2. The TGS program sample was received at a CDC laboratory for rapid virus isolation and sequencing confirmation. BA.3.2 has since been detected in multiple TGS program specimens. NWSS and WastewaterSCAN have detected BA.3.2 in hundreds of wastewater samples across geographically diverse areas, with recurring detections in some catchment areas, indicating ongoing circulation. In most states, detections of the variant in wastewater occurred many weeks before detection in clinical specimens from patients; therefore, wastewater surveillance served as an effective early warning system for this SARS-CoV-2 variant. Limited detection in clinical specimens in the national SARS-CoV-2 genomic surveillance program likely reflects a decline in sequencing submissions since 2023. Isolates identified through health care–based surveillance, particularly those from hospitals, are obtained from patients who are more likely to have specimens collected and whose specimens are more likely to be sequenced. However, detection of BA.3.2 isolates from hospitalized patients does not necessarily indicate that the variant causes more severe disease, nor does it establish any association with risk factors, especially because, to date, this group of patients represents a small case series.

### Limitations

The findings of this report are subject to at least five limitations. First, variations in international sequencing and reporting capacity might limit the ability to track and monitor the geographic spread of BA.3.2. Second, variable timing between specimen collection and submission of genomic sequences limits real-time detection of emerging variants, which might delay public health responses. Third, the absence of standardized international methods for submitting genomic sequences to public repositories, especially for wastewater surveillance, limits data timeliness and comparability and might have affected detection of variants. Fourth, the decrease in the number of available U.S. sequences and specimens has reduced the sensitivity of genomic surveillance, delaying the identification of emerging SARS-CoV-2 variants and possibly underestimating the extent of geographic spread. Finally, although genomic differences in BA.3.2 and its geographic spread warrant continued monitoring, the impact of these findings on human health outcomes (e.g., illness severity and health care system impact) remains to be determined.

### Implications for Public Health Practice

The 2025–2026 COVID-19 vaccines (LP.8.1-adapted mRNA formulation and JN.1-adapted protein formulation) provide protection against the currently circulating predominant U.S. variants. However, in laboratory studies, the recently emerged BA.3.2 strain efficiently evades antibodies, likely because of spike protein mutations, highlighting the need for ongoing genomic surveillance and observational evaluations of vaccine and antiviral effectiveness. Although widespread infection- and vaccine-conferred immunity have decreased rates of severe COVID-19 over time, the public health impact of COVID-19 is still considerable: an estimated 390,000–550,000 hospitalizations and 45,000–64,000 deaths occurred during the 2024–25 respiratory virus season (Preliminary Estimates of COVID-19 Burden for 2024–2025 | COVID-19 | CDC). New SARS-CoV-2 variants with substantial capacity to evade immunity from previous infections or vaccines could be associated with seasonal increases in COVID-19 activity. Robust surveillance data will continue to guide CDC’s preparedness, ensure timely responses to emerging SARS-CoV-2 variants, and guide decision-making on COVID-19 vaccine composition updates.

## Footnotes

* Includes GISAID sequences and metadata through February 11, 2026. https://doi.org/10.55876/gis8.260223rg

† The TGS program collects nasal swab samples from arriving international travelers and wastewater from airplanes. Wastewater from individual airplanes and combined wastewater from multiple airplanes are collected from an airport triturator.

§ WastewaterSCAN is a partnership among Stanford University, Emory University, and Verily that monitors infectious diseases through municipal wastewater systems.

¶ The initial variant detection and relative abundance in wastewater were inferred through deconvolution using Freyja, with BA.3.2-specific mutation co-occurrences further verified using CoOccurence adJusted Analysis and Calling.

** 45 C.F.R. part 46.102(l)(2), 21 C.F.R. part 56; 42 U.S.C. Sect. 241(d); 5 U.S.C. Sect. 552a; 44 U.S.C. Sect. 3501 et seq.

†† A self-collected nasal swab sample obtained by the TGS program and identified as SARS-CoV-2 lineage BA.3.2 was received at CDC for virus isolation. The specimen was cultured in VeroE6 cells overexpressing TMPRSS2/ACE2, harvested, quantified, tested for presence of multiple potential co-infecting respiratory pathogens, and sequenced for characterization. Isolate name: SARS-CoV-2/Human/USA/NY-GBW-GKISBBBI08250/2025. BioSample SRA Artifactid: SAMN54262640 SRR36563121 85617–1453-B1.

§§ Samples were collected on November 5, 2025 (triturator), December 7, 2025 (airplane), and December 12, 2025 (triturator).

¶¶ California (two), Connecticut (six), Florida (two), Hawaii (two), Idaho (one), Illinois (one), Maine (19), Maryland (six), Massachusetts (nine), Missouri (one), New Hampshire (17), New Jersey (three), Nevada (one), New York (seven), Pennsylvania (four), Rhode Island (27), South Carolina (one), Texas (one), Utah (three), Vermont (one), Virginia (one), and Wyoming (two).

*** California (three), Louisiana (one), Maine (one), Michigan (one), Massachusetts (five), and Ohio (four).
